# Multiple Host Kinases Contribute to Akt Activation during *Salmonella* Infection

**DOI:** 10.1371/journal.pone.0071015

**Published:** 2013-08-22

**Authors:** Bernhard Roppenser, Hyunwoo Kwon, Veronica Canadien, Risheng Xu, Peter N. Devreotes, Sergio Grinstein, John H. Brumell

**Affiliations:** 1 Cell Biology Program, Hospital for Sick Children, Toronto, Ontario, Canada; 2 Department of Molecular Genetics, University of Toronto, Toronto, Ontario, Canada; 3 Department of Neuroscience, Johns Hopkins Medical School, Baltimore, Maryland, United States of America; 4 Department of Cell Biology, Johns Hopkins University School of Medicine, Baltimore, Maryland, United States of America; 5 Institute of Medical Science, University of Toronto, Toronto, Ontario, Canada; 6 Department of Biochemistry, University of Toronto, Toronto, Ontario, Canada; 7 Sickkids IBD Centre, Hospital for Sick Children, Toronto, Ontario, Canada; Indian Institute of Science, India

## Abstract

SopB is a type 3 secreted effector with phosphatase activity that *Salmonella* employs to manipulate host cellular processes, allowing the bacteria to establish their intracellular niche. One important function of SopB is activation of the pro-survival kinase Akt/protein kinase B in the infected host cell. Here, we examine the mechanism of Akt activation by SopB during *Salmonella* infection. We show that SopB-mediated Akt activation is only partially sensitive to PI3-kinase inhibitors LY294002 and wortmannin in HeLa cells, suggesting that Class I PI3-kinases play only a minor role in this process. However, depletion of PI(3,4) P_2_/PI(3–5) P_3_ by expression of the phosphoinositide 3-phosphatase PTEN inhibits Akt activation during *Salmonella* invasion. Therefore, production of PI(3,4) P_2_/PI(3–5) P_3_ appears to be a necessary event for Akt activation by SopB and suggests that non-canonical kinases mediate production of these phosphoinositides during *Salmonella* infection. We report that Class II PI3-kinase beta isoform, IPMK and other kinases identified from a kinase screen all contribute to Akt activation during *Salmonella* infection. In addition, the kinases required for SopB-mediated activation of Akt vary depending on the type of infected host cell. Together, our data suggest that *Salmonella* has evolved to use a single effector, SopB, to manipulate a remarkably large repertoire of host kinases to activate Akt for the purpose of optimizing bacterial replication in its host.

## Introduction

*Salmonella enterica* serovar Typhimurium ( *S* *. Typhimurium* ) is a major cause of food poisoning worldwide and infections can be lethal in young children or immunocompromised hosts [1,2]. These bacteria have the ability to invade host cells and grow intracellularly. To accomplish this, *S* *. Typhimurium* uses type 3 secretion systems (T3SS) to translocate effector proteins directly into host cells [3]. The T3SS effectors activate signal transduction pathways leading to actin rearrangements that drive internalization of the bacteria into membrane-bound compartments, known as Salmonella-containing vacuoles (SCVs), within which the bacteria can survive and replicate [4,5]. One T3SS effector, SopB, is known to contribute to *S* *. Typhimurium* invasion [6]. SopB also contributes to other important phenotypes associated with infection, including production of proinflammatory cytokines [7], controlling SCV maturation [8], intracellular positioning of the SCV [9], blocking apoptosis [10], intracellular bacterial replication [8] and induction of epithelial-mesenchymal transition [11].

SopB is an important modulator of signal transduction pathways in host cells during infection and, together with two other T3SS effectors –SopE and SopE2- SopB influences members of the small Rho GTPase family [6]. It was demonstrated that SopB activates RhoG via its guanine nucleotide exchange factor SGEF, leading to actin rearrangements that promote bacterial invasion [7]. In addition, SopB can activate the pro-survival kinase Akt, preventing apoptosis in infected cells [10,12]. SopB was found to be solely responsible for Akt activation and subsequent inhibition of caspase-3 activity, protecting infected epithelial cells from apoptosis [10]. Thus, SopB acts as a pro-survival factor, preventing apoptosis and allowing the generation of a replicative niche in infected cells. Yet, the mechanisms by which SopB promotes Akt activation are unclear.

SopB contains a catalytic domain with phosphoinositide phosphatase activity that is essential for all of its associated phenotypes [6,13,14]. It has broad substrate specificity *in vitro*, but *in vivo* substrates have been the subject of controversy and the mechanisms by which it acts are unclear. The main substrate of SopB during infection is PI(4,5) P_2_, leading to the generation of PI(5) P [15,16]. Paradoxically, despite the fact that *in vitro* SopB can dephosphorylate PI(3,4) P_2_ and PI(3–5) P_3_, Mallo et al. showed that SopB is actually required and responsible for the accumulation of these lipids at invasion ruffles [15]. In the canonical pathway of Akt activation, e.g. during growth factor stimulation, Class I PI3-kinase generates PI(3,4) P_2_ and PI(3–5) P_3_ in the plasma membrane, where Akt binds and then becomes phosphorylated on Thr308 and Ser473 by PDK1 and mTORC2 complex, respectively [17–19]. However, the equivalent PI-accumulation during *Salmonella* invasion was found to be resistant to the PI3-kinase inhibitor LY294002 [15], suggesting that in this instance PI(3,4) P_2_ and PI(3–5) P_3_ are generated by a Class I PI3-kinase independent mechanism. Indeed, siRNA-mediated depletion of Class I PI3-kinase regulatory subunits p85α and p85β had no inhibitory effect on SopB-mediated Akt activation [20]. Despite this, Steele-Mortimer and colleagues showed that PDK1 and mTORC2 are required for SopB-mediated Akt activation [20]. These authors concluded that SopB activates Akt by a mechanism independent of Class I PI3-kinase that nevertheless incorporates some elements of the canonical pathway. Still, the role of PI(3,4) P_2_ and PI(3–5) P_3_ in Akt activation by SopB has not been tested directly.

The mechanisms that regulate Akt activation during *Salmonella* infection remain unclear. Here, we show that localized PI(3,4) P_2_ and PI(3–5) P_3_ production at invasion ruffles is responsible for Akt activation. Furthermore, we show that SopB exploits several different host kinases, depending on the infected cell type, to activate Akt during invasion. Our studies provide important novel insight into bacterial modulation of host kinase activity that can lead to the establishment of an intracellular replicative niche.

## Materials and Methods

### Plasmids and bacterial strains

A plasmid encoding untagged SopB was previously described [21]. GFP-SopB was previously described [22] and provided by D. Zhou (Purdue University). pEGFP-C1 was from Clontech. PTEN-A4-YFP was described previously [23]. GFP-Akt was a gift from M. Molina (Universidad Complutense de Madrid). Plasmids expressing His-SopB and catalytically inactive His-SopB-C460S [13] were provided by B. Finlay (University of British Columbia). Wild type *Salmonella enterica* serovar Typhimurium ( *S* *. Typhimurium* ) SL1344 [24] and isogenic *ΔsopB* mutant [12] strain were used in this study.

### Antibodies and Reagents

Rabbit polyclonal antibodies to Ser473-phosphorylated-Akt and pan-Akt were purchased from Cell Signaling. Vps34 antibody was described elsewhere [25] and provided by J. Backer (Albert Einstein College of Medicine). Rabbit polyclonal antibody to *S* *. Typhimurium* O antiserum Group B was from Difco. Murine monoclonal anti-GFP antibody was obtained from Invitrogen. Mouse anti-GAPDH antibody was from Milipore. Secondary antibodies used for immunofluorescence study and Phalloidin were Alexa-conjugated and purchased from Molecular Probes. Recombinant human EGF and Wortmannin were purchased from Invitrogen. LY294002 was from Cell Signaling.

### Cell Culture and bacterial infections

HeLa and MEFs were grown in high-glucose DMEM (Hyclone) supplemented with 10% FBS (Wisent) at 37°C in 5% CO_2_. IPMK knockout MEFs were previously described [26]. IPMK knockout embryonic stem cells were previously described [27] and maintained in high-glucose DMEM (Hyclone) supplemented with 12.5% ES cell-qualified FBS (Gibco), 1x Penicillin/Streptomycin (Wisent), 100 µM MEM non-essential amino acids (Wisent), 55 µM 2-mercaptoethanol (Gibco) and Esgro (Chemicon International) at 37°C in 5% CO_2_. Cells were transfected with Genejuice (Oncogene Research Products) or Amaxa (Lonza) according to manufacturer’s instructions and used 16 hours after transfection.

*S* *. Typhimurium* infections were carried out as described previously [28]. Briefly, bacteria were grown overnight shaking at 37°C and then subcultured in LB without antibiotics for 3 hours. Bacterial inoculum was prepared by pelleting 1 mL of the subculture at 10,000 x g for 2 min, washed once and diluted 1:100 in PBS. PI3-kinase inhibitors were added 30 min prior to the infection and maintained throughout the experiment.

### siRNA/esiRNA oligonucleotides and transfections

Control siRNA and those directed against Class II PI3-kinases and IPMK were obtained from Sigma. Vps34 siRNA was described elsewhere [15]. esiRNA against all kinases were obtained from the SIDNET facility at the Hospital for Sick Children. Cells grown in 24-well plates were transfected with 200 nM of individual siRNAs or 200 ng of individual esiRNAs using Lipofectamine® RNAiMAX transfection reagent (Life Technologies) and used for experiments 48 hours after transfection. To confirm knockdown, RNA was isolated with an RNeasy Mini kit (Qiagen) and cDNA was transcribed with the iScript cDNA synthesis kit (BioRad). A qPCR reaction was performed using the SSO advanced SYBR green mix (BioRad).

### Immunofluorescence and Western Blot analysis

Fixed cells were immunostained as described [29]. To differentiate between intra- and extracellular bacteria, cells were immunostained before permeabilization with saponin. Images were acquired with a Quorum spinning disk microscope with a 63 x oil immersion objective (Leica DMIRE2 inverted fluorescence microscope equipped with a Hamamatsu Back-Thinned EM-CCD camera, spinning disk confocal scan head). Quantification of samples was done with an epifluorescence microscope (DMIRE2; Leica) equipped with a 100x/NA 1.4 oil objective (Plan Apochromat; Leica).

For Western Blot analysis, cells were washed three times with PBS and then lysed with RIPA buffer. Samples were separated on 10% SDS-PAGE gels, transferred to PVDF-membranes and blocked in 5% milk for 1 hour. Primary and Secondary antibodies were incubated in 2% BSA solution. Bands were detected using the ECL detection system (GE Healthcare).

### Calculation of the relative phospho-Akt expression level

Intensity of the phospho-Akt protein band on a Western blot was estimated using ImageJ software. In order to account for potential differences in the total amount of proteins from one sample to another, intensity of the phospho-Akt band was divided by that of the total Akt band. This quotient is the relative phospho-Akt expression level. In an experiment, all relative p-Akt expression levels were normalized to that of the control sample.

### In vitro dephosphorylation assay and thin layer chromatography

His-SopB and His-SopB-C460S were recombinantly expressed in *E. coli* BL21 and affinity purified with HisTrap Ni-Sepharose columns according to Manufacturer’s instructions (GE Healthcare). BODIPY® FL PI(4,5) P_2_ C6 and BODIPY® FL PI(3–5) P_3_ C6 were from Cedarlane. To make liposomes, the required amount of stock lipids (in chloroform) was dried under N_2_ and then resuspended in 50mM Tris buffer. Liposomes were incubated with recombinant SopB and then separated on 250 µM silica gel TLC plates (Whatman) with a resolving solution of 2-Propanol, NH_4_OH and H_2_O (65:20:15). Lipids were visualized with a Storm 840 chemiluminescence imager system (Molecular Dynamics).

## Results

### SopB-mediated Akt activation at *Salmonella* invasion ruffles is PI (3,4) P_2_/PI (3–5) P_3_-dependent

**Figure 1:**
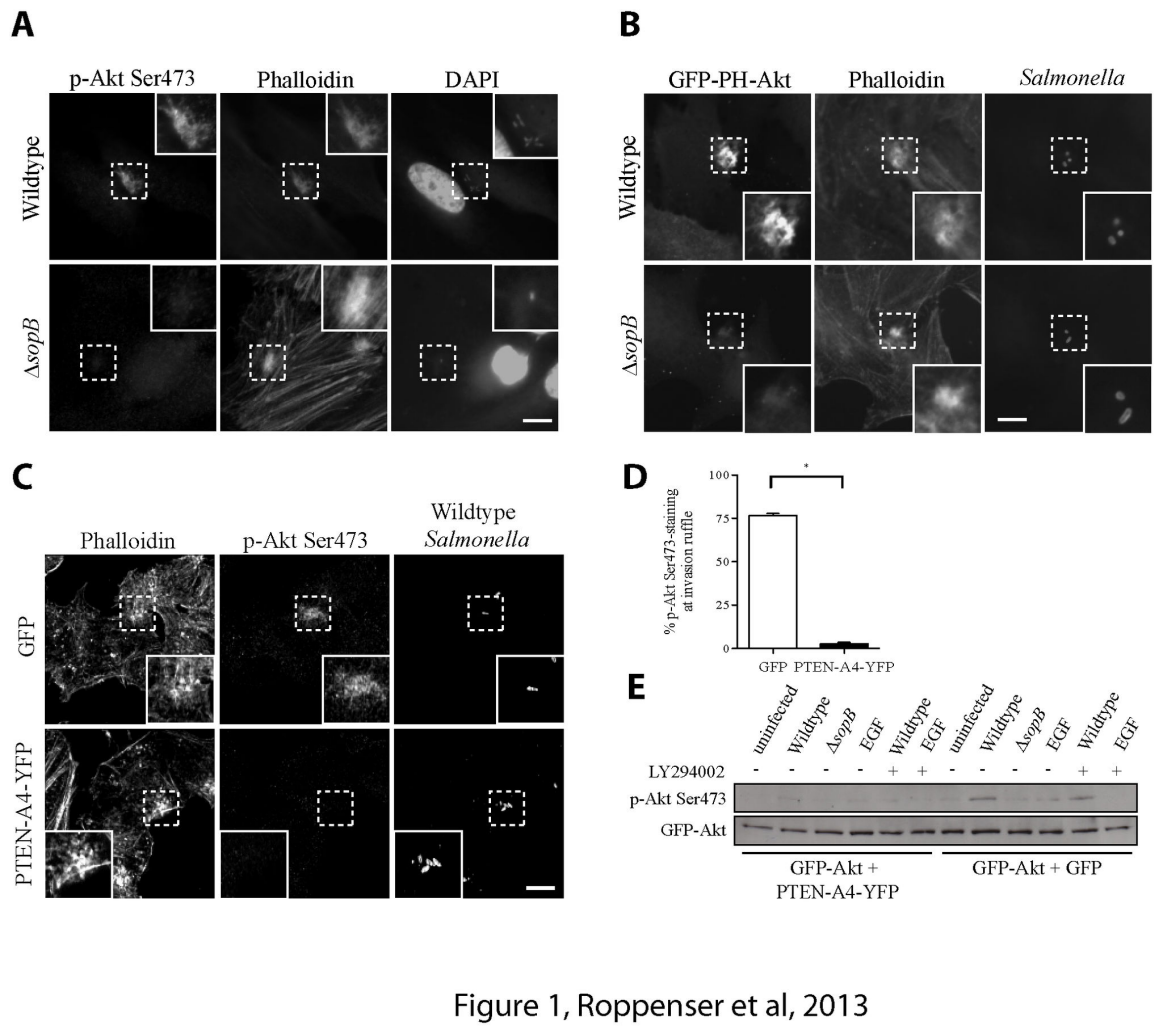
SopB-mediated Akt activation at *Salmonella* invasion ruffles is PI(3,4) P_2_/PI(3–5) P_3_-dependent. (A) HeLa cells were infected with wild type or Δ*sopB* mutant *S* *. Typhimurium* and fixed at 30 min p.i. Cells were examined by epifluorescence microscopy after co-staining for activated Akt with a phospho-specific (Ser473) antibody, actin with fluorescently-labeled phalloidin and bacteria with DAPI. Insets are enlarged from boxed areas. (B) HeLa cells were transiently transfected with GFP-PH-Akt for 16 h prior to *Salmonella* infection. Cells were infected and fixed as in A. Cells were co-stained for actin with fluorescently-labeled phalloidin and bacteria with a polyclonal antibody to *S* *. Typhimurium* . Insets are enlarged from boxed areas. (C) HeLa cells were transiently transfected with GFP or PTEN-A4-YFP for 16 h prior to *Salmonella* infection. Cells were infected with wild type *S* *. Typhimurium* and fixed as in A. Cells were co-stained for actin with fluorescently-labeled phalloidin, Akt with a phospho-specific (Ser473) antibody and bacteria with a polyclonal antibody to *S* *. Typhimurium* . Insets are enlarged from boxed areas. Size bars, 10 µm. (D) The percentage of phospho-Akt^+^ *Salmonella* invasion ruffles from C was quantified (n ≥ 50). Averages ± SD for three separate experiments are shown. Asterisk indicates that the percent value is significantly different from the control (P < 0.001) as determined by one-way ANOVA analysis. (E) HeLa cells were transiently transfected with GFP-Akt and PTEN-A4-YFP or GFP for 16 h prior to infection. Cells were uninfected, infected as in A, or incubated with 100 ng/mL EGF for 5 min. Where indicated, cells were treated with 100 µM LY294002 for 30 min prior to infection. Akt activation was determined by immunoblotting the cell lysates with a phospho-specific anti-Ser473 Akt antibody. GFP antibodies were used to ensure equal Akt transfection.

SopB is necessary for the activation of Akt during *Salmonella* invasion, as judged by western blotting with phospho-specific antibodies that recognize the active form of Akt, which is phosphorylated at Serine 473 (pAkt-Ser473) [12]. Cooper et al. previously showed that phosphorylated Akt is recruited to *Salmonella* ruffles in SopB-dependent manner [20]. Consistent with this earlier finding, we observed accumulation of pAkt-Ser473 at the actin-rich bacterial invasion site, which was visualized by phalloidin staining (Figure 1A). Such accumulation of pAkt-Ser473 at invasion sites was not observed following infection by a bacterial SopB-deficient (Δ*sopB*) mutant (Figure 1A). We also observed that cells transfected with plasmids expressing SopB showed significant increase in Akt activation compared to control (GFP-transfected) cells (Figure S1). Thus, SopB is both necessary and sufficient for Akt activation.

We previously showed that SopB leads to the formation of PI(3,4) P_2_ and PI(3–5) P_3_ at bacterial invasion sites [15]. Visualization of PI(3,4) P_2_/PI(3–5) P_3_ is accomplished by expression of GFP-PH-Akt, a fluorescent probe for these phosphoinositides. Wild type bacteria led to recruitment of GFP-PH-Akt to invasion ruffles, while a bacterial Δ*sopB* mutant did not, consistent with our previous studies [15] (Figure 1B). Since formation of PI(3,4) P_2_ and PI(3–5) P_3_ at membranes is required for canonical Akt activation, we tested if these phosphoinositides are required for SopB-dependent Akt activation by inhibiting their accumulation at invasion sites. This was accomplished by expression of PTEN, a PI-3- phosphatase that specifically dephosphorylates PI(3,4) P_2_ and PI(3–5) P_3_. Expression of YFP-tagged PTEN-A4 (with 4 alanine substitutions S380A, T382A, T383A, and S385A) has been successfully used to block accumulation of these PIs [23]. Mutations in this construct are at the regulatory phosphorylation sites at the C-terminus of PTEN, making the resultant protein more active by targeting it to the plasma membrane [23]. After transfection of this modified PTEN construct, SopB-mediated Akt activation was almost completely abolished at invasion sites, as determined by immunofluorescence (Figure 1C, D). A similar decrease in Akt activation can be seen by immunoblotting (Figure 1E). Together, these results suggest that PI(3,4) P_2_/PI(3–5) P_3_ production is required for Akt activation by SopB.

### SopB-Mediated Akt Activation in HeLa Cells Is Only Partially Sensitive to PI3-Kinase Inhibitors LY294002 and Wortmannin

**Figure 2:**
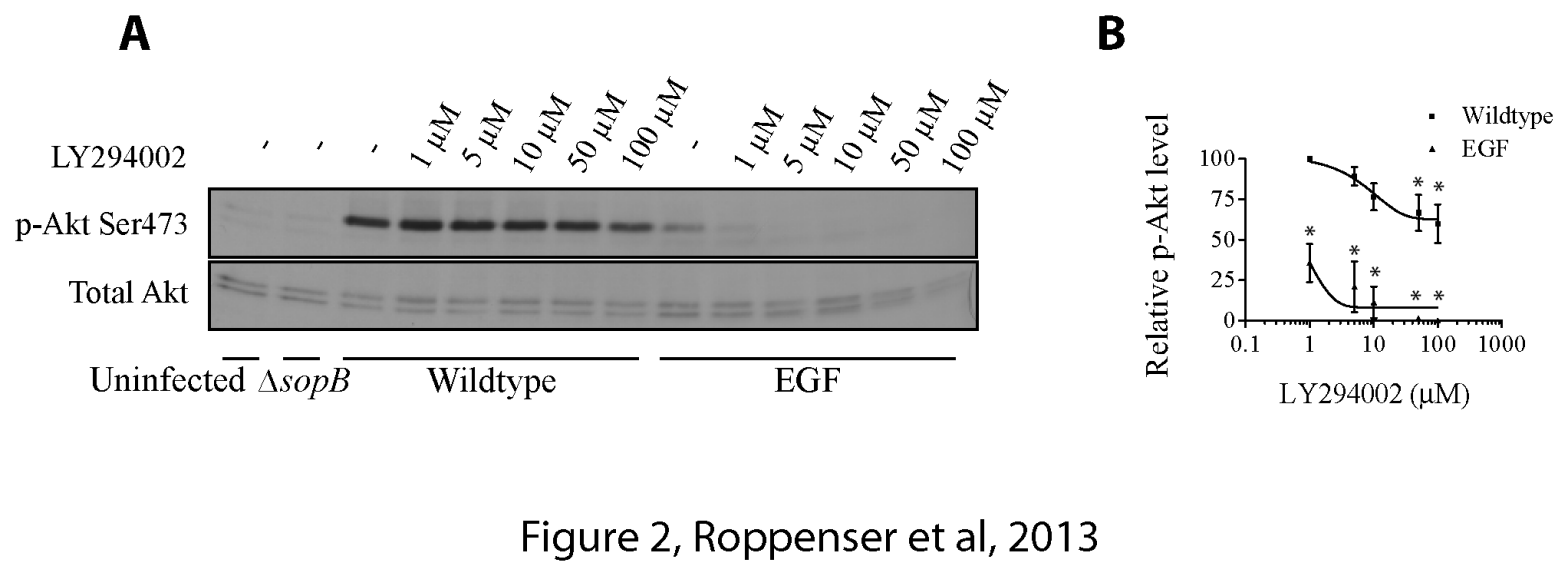
SopB-mediated Akt activation in HeLa cells is only partially sensitive to PI3-Kinase inhibitor LY294002. (A) HeLa cells were treated with different concentrations of LY294002 (1 µM to 100 µM) for 30 min. Cells were then infected with wild type *S* *. Typhimurium* for 30 min or incubated with 100 ng/mL EGF for 5 min. As controls, cells were either uninfected or infected with Δ*sopB* mutant *S* *. Typhimurium* for 30 min. Akt activation was determined by immunoblotting the cell lysates with a phospho-specific anti-Ser473 Akt antibody. Pan-Akt antibodies were used to ensure equal protein loading. (B) Western blot results from A were analyzed by estimating the intensities of protein bands with the ImageJ software. Shown on the graph are the relative and normalized expression levels of phospho-Akt ± SD induced by wild type *S* *. Typhimurium* or EGF for three separate experiments, calculated as outlined in the Materials and Methods. Asterisks indicate that the percent value is significantly different from the control (P < 0.05).

PI3-kinases, which phosphorylate PIs at the 3-position of the inositol ring, can be divided into three main classes depending on their substrate specificity [30]. We tested all 3 classes of PI3-kinases for their ability to influence SopB-mediated Akt activation. Class I PI3-kinases can be effectively inhibited by the PI3-kinase inhibitors LY294002 and Wortmannin. HeLa cells were treated with varying concentrations of LY294002 (Figure 2) or Wortmannin (Figure S2), then infected with wild type *Salmonella* . Akt activation was measured in cell lysates with anti-pAkt-Ser473 antibodies. Addition of low concentrations of LY294002 did not significantly impair SopB-mediated Akt activation during infection. However, higher concentrations, e.g. 50-100 µM, LY294002 significantly reduced the Akt activation by approximately 25% compared to the control. Wortmannin showed a very similar impact on Akt activation. In contrast, Akt activation by EGF was abolished by low concentrations of either inhibitor. This indicates that Class I PI3-kinases, which are normally inhibited by LY294002 or Wortmannin, play a minor role in the SopB-mediated Akt activation in HeLa cells.

### Class II PI3-Kinase beta isoform (PI3K-C2β) contributes to SopB-mediated Akt activation

**Figure 3:**
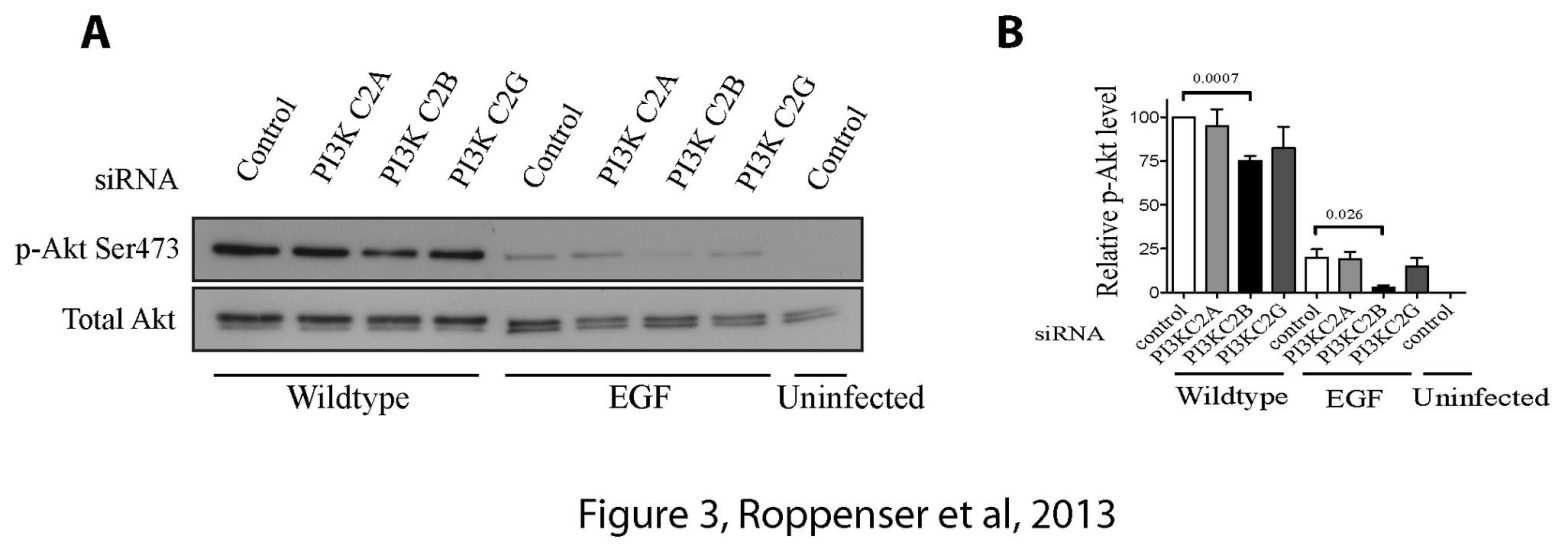
Class II PI3-Kinase beta isoform (PI3K-C2β) plays a role in SopB-mediated Akt activation. (A) HeLa cells were treated with control, PI3K-C2A, PI3K-C2B or PI3K-C2G siRNA for 48 h. Cells were then infected with wild type *S* *. Typhimurium* for 30 min. As controls, cells were uninfected or incubated with 100 ng/mL EGF for 5 min. Akt activation was determined by immunoblotting the cell lysates with a phospho-specific anti-Ser473 Akt antibody. Pan-Akt antibodies were used to ensure equal protein loading. (B) Western blot results from A were analyzed by estimating the intensities of protein bands with the ImageJ software. Shown on the graph are the relative and normalized expression levels of phospho-Akt ± SD induced by wild type *S* *. Typhimurium* or EGF for three separate experiments, calculated as outlined in the Materials and Methods. The p-values from one-way ANOVA analysis are shown.

Class II PI3-kinases are less sensitive to PI3-kinase inhibitors [31] and Class II-mediated Akt activation has been shown to be required for glucose-stimulated insulin secretion [32]. Therefore, we targeted expression of all Class II PI3-kinase isoforms using siRNA and analyzed Akt activation by *Salmonella* . Knockdown of PI3K-C2β led to a significant decrease in SopB-dependent Akt activation, while that of other Class II PI3-kinase isoforms was without effect (Figure 3). Though silencing PI3K-C2β reduced Akt phosphorylation, this kinase seemed to only have a partial role in Akt activation in HeLa cells, indicating that other kinases are also involved.

### Class III PI3-Kinase Vps34 does not play a role in SopB-mediated Akt activation

**Figure 4:**
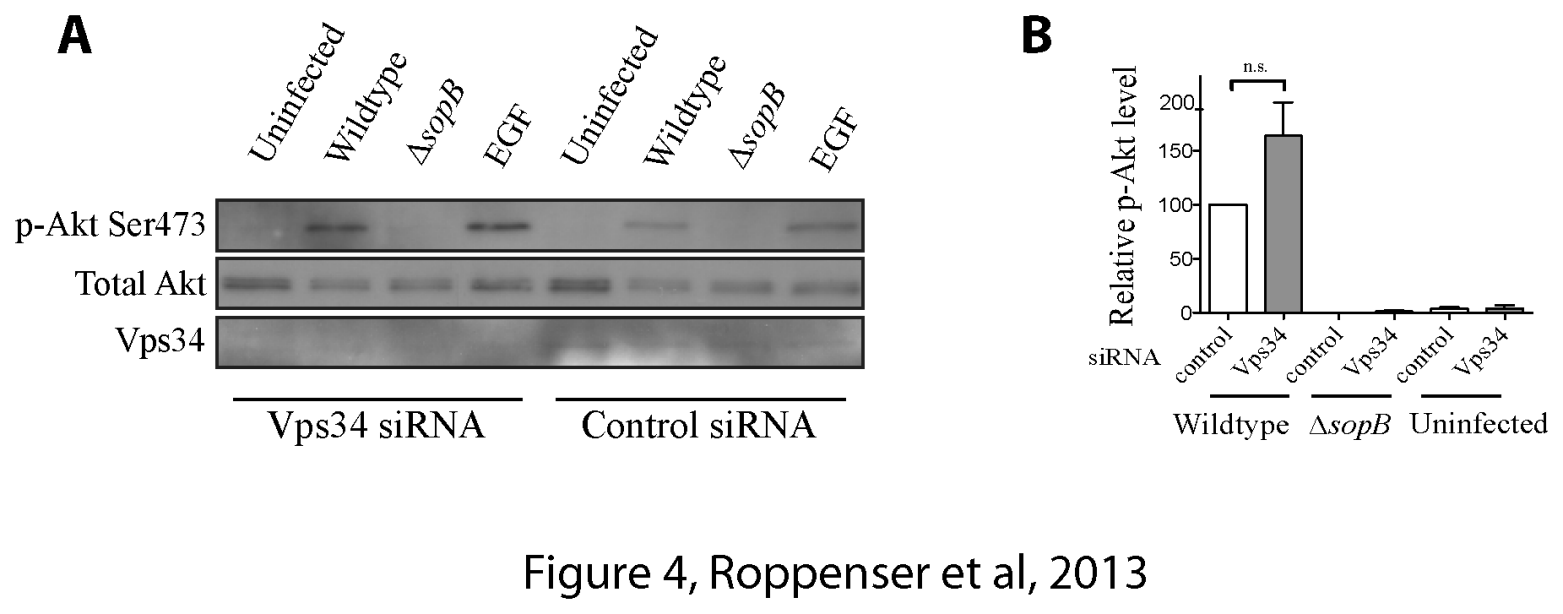
Class III PI3-Kinase does not contribute to SopB-mediated Akt activation. (A) HeLa cells were treated with control or Vps34 siRNA for 48 h. Cells were infected with wild type or ΔSopB mutant *S* *. Typhimurium* for 30 min. As controls, cells were uninfected or incubated with 100 ng/mL EGF for 5 min. Akt activation was determined by immunoblotting the cell lysates with a phospho-specific anti-Ser473 Akt antibody. Pan-Akt antibodies were used to ensure equal protein loading. Anti-Vps34 antibody was used to verify the knockdown of Vps34 in cells that were treated with siRNA. (B) Western blot results from A were analyzed by estimating the intensities of protein bands with the ImageJ software. Shown on the graph are the relative and normalized expression levels of phospho-Akt ± SD for three separate experiments, calculated as outlined in the Materials and Methods. Statistical significance was assessed by one-way ANOVA analysis.

Class III PI3-kinase Vps34 mediates phosphorylation of PI, generating PI3P. Previous studies suggested that phosphorylation of PI3P can lead to production of PI(3,4) P_2_ and PI(3–5) P_3_ [33]. Therefore, we tested the role of Vps34 in *Salmonella* -induced Akt activation, targeting its expression with siRNA. Knockdown of Vps34 did not impair SopB-dependent activation of Akt, indicating that the Class III PI3-kinase does not contribute to SopB-mediated Akt activation (Figure 4).

### SopB does not directly generate PI (3–5) P_3_ by phosphotransfer

Since the canonical PI3-kinases cannot fully account for Akt activation during *Salmonella* invasion, we considered other possible mechanisms by which SopB could lead to the production of PI(3,4) P_2_ and PI(3–5) P_3_. Because some bacterial proteins can catalyze phosphotransfer reactions between proteins [34], we considered the possibility that SopB might be mediating phosphotransfer reactions between phosphoinositides. Such a catalytic activity for SopB has not been formally tested *in vitro*. To determine if SopB has unrecognized catalytic activity and is by itself sufficient to induce PI(3–5) P_3_ formation through a phosphotransfer reaction, we performed an *in vitro* assay with recombinant SopB and fluorescently tagged PIs. In our assays, SopB induced a robust dephosphorylation of PI(4,5) P_2_ and PI(3–5) P_3_, generating phosphatidylinositol monophosphates (Figure S3). However, formation of PI(3–5) P_3_ by SopB was not detected in our assay. Our studies therefore indicate that SopB does not directly mediate production of PI(3–5) P_3_ during infection, but rather modulates unknown host factors to generate this phosphoinositide by a non-canonical pathway.

### A kinase screen reveals that IPMK plays a role in SopB-mediated Akt activation in HeLa cells

**Figure 5:**
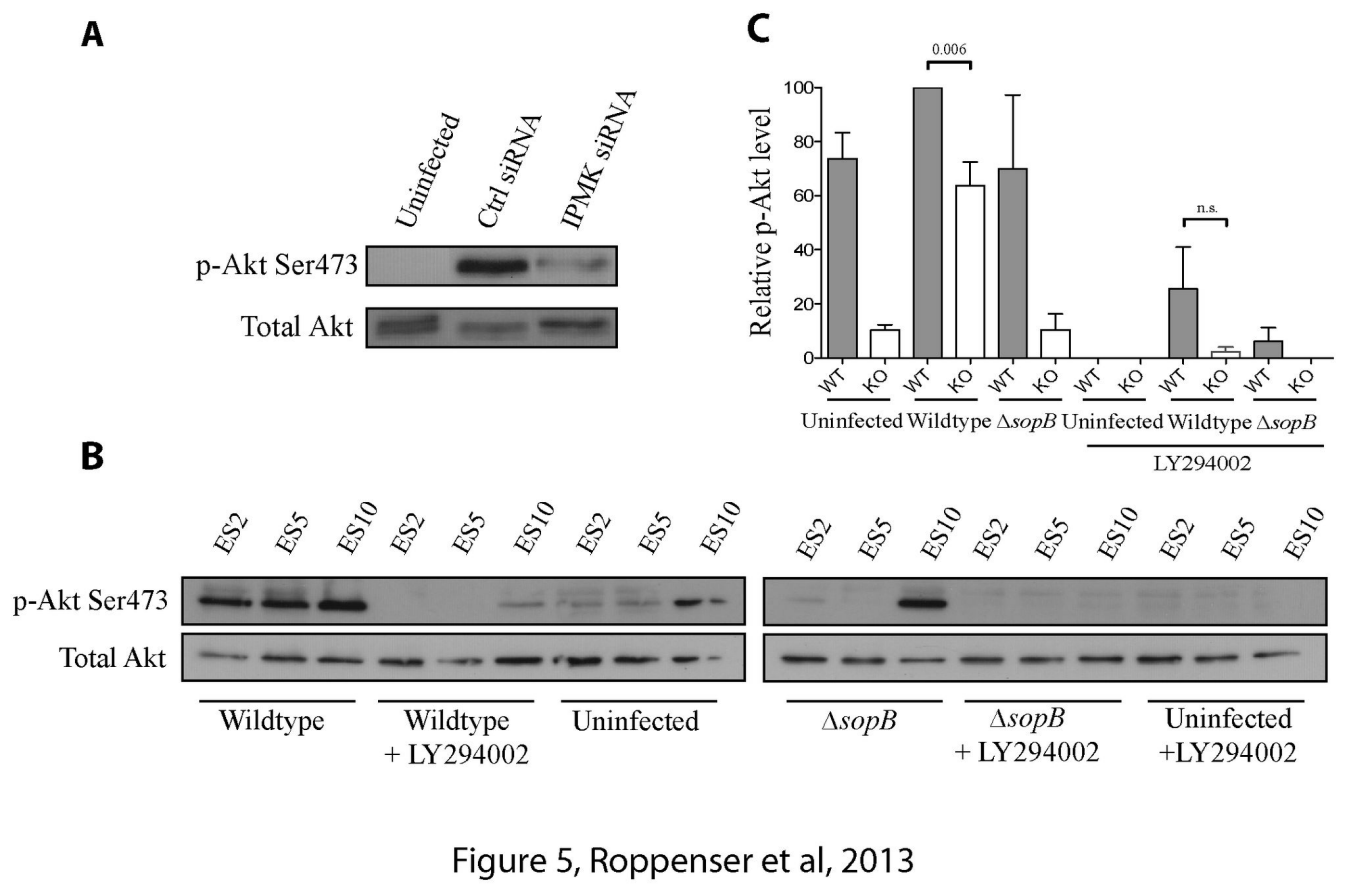
IPMK plays a role in SopB-mediated Akt activation. (A) HeLa cells were treated with control or IPMK siRNA for 48 h. Cells were infected with wild type *S* *. Typhimurium* for 30 min. As a control, cells were uninfected. Akt activation was determined by immunoblotting the cell lysates with a phospho-specific anti-Ser473 Akt antibody. Pan-Akt antibodies were used to ensure equal protein loading. (B) Wild type (ES10) or IPMK knockout (ES2 and ES5) embryonic stem cells were infected with wild type or ΔSopB mutant *S* *. Typhimurium* for 30 min. As a control, cells were uninfected. Where indicated, cells were treated with 100 µM LY294002 for 30 min. prior to infection. Akt activation was determined by immunoblotting the cell lysates with a phospho-specific anti-Ser473 Akt antibody. Pan-Akt antibodies were used to ensure equal protein loading. (C) Western blot results of ES10 and ES5 from B were analyzed by estimating the intensities of protein bands with the ImageJ software. Shown on the graph are the relative and normalized expression levels of phospho-Akt ± SD for three separate experiments, calculated as outlined in the Materials and Methods. The p-values from one-way ANOVA analysis are shown.

In search for the non-canonical host kinase(s) involved in SopB-mediated Akt activation, we performed an esiRNA screen of 778 mammalian kinases. The results of our screen are shown in Table 1. Many of the kinases identified have been previously implicated in regulating Akt activity (highlighted in yellow; see references), supporting the validity of our screen. The top ‘hit’ in our screen was inositol polyphosphate multikinase (IPMK). SopB-mediated Akt activation was impaired by as much as 91% when IPMK expression was targeted by esiRNA (Table 1). We also targeted expression of IPMK using siRNA for comparison. siRNA-mediated silencing of IPMK expression similarly impaired Akt activation, providing additional evidence for its role in HeLa cells (Figure 5A).

**Table 1:** Kinases that play a role in Akt activation during *Salmonella* infection.

**Gene symbol**	**% Activation compared to control**	**References**	**Gene symbol**	**% Activation compared to control**	**References**
IPMK	8.47	[26]	PRKCN	39.76	-
CHKA	11.09	[40]	PDXK	39.76	-
HCK	12.03	[41]	PRKCD	44.74	-
NADK	13.15	-	PRKAR1A	45.86	-
MAST2	15.18	-	ULK2	47.05	-
MINK	16.77	-	PRKR	48.05	-
SSTK	18.97	-	DAPK2	56.70	-
PNKP	19.11	-	FASTK	60.99	-
MAP3K2	20.76	-	ADCK4	62.56	-
MAP3K9	21.09	-	PAK1	62.88	[44]
CDC2	23.06	-	ULK3	63.49	-
DGUOK	23.32	-	MARK3	64.04	-
ACK1	25.33	[42]	LCK	64.21	[45]
SNRK	27.46	-	ILK	67.01	[46]
ERK8	28.08	-	PIK3C2A	70.06	-
AGK	28.09	-	SRC	71.09	[47]
CDKL5	28.25	-	PANK1	72.28	-
PFKFB1	29.10	-	BLNK	73.06	-
LYK5	30.26	-	STK11	73.36	-
MIDORI	30.80	-	PRKCH	74.43	-
AK1	32.42	-	CDC2L2	75.12	-
HK3	36.07	-	AURKC	79.06	-
BLK	36.77	-	PRKCQ	79.72	[48]
PLK1	38.36	[43]	MGC8407	80.59	-
PRKACG	39.20	-	IKBKE	83.02	[49]
CKB	39.33	-	PIP5K1A	89.48	-

HeLa cells were treated with esiRNAs to mammalian kinases (778 were tested individually) for 48 h. Cells were then infected with wild type *S* *. Typhimurium* for 30 min. Akt activation was determined by immunoblotting the cell lysates with a phospho-specific anti-Ser473 Akt antibody. GAPDH antibodies were used to ensure equal protein loading. Relative phospho-Akt levels were determined using ImageJ software. Activation of Akt shown in the table was normalized to that of control esiRNA-treated sample. Among the listed 52 most highly ranked kinases, of which their knockdowns resulted in the most significant decrease in Akt activation, those with indicated references have been previously implicated in the positive regulation of Akt.

To further explore the role of IPMK in SopB-mediated Akt activation, we used two different cell types lacking IPMK. In IPMK knockout embryonic stem (ES) cells [27], we observed a significant decrease in Akt activation upon *Salmonella* infection, compared to wild type control cells (Figure 5B and C). We also examined mouse embryonic fibroblast cells (MEFs) lacking IPMK [35]. In contrast to ES cells, we did not see a decrease in Akt activation in IPMK^-/-^ MEFs when infected with wild type Salmonella compared to WT MEFs (Figure S4). However, when we treated the ES and MEF cells with the PI3-kinase inhibitor LY294002, we saw a significant decrease in SopB-mediated Akt activation, suggesting that Class I PI3-kinase may play a dominant role in these cell types unlike in HeLa cells. Together, our studies demonstrate that IPMK contributes to Akt activation in HeLa cells, but has a minor or negligible role in other tested cell types.

## Discussion

Recently, we showed that PI(3,4) P_2_ and PI(3–5) P_3_ are generated at invasion ruffles in a SopB-dependent manner [15]. In our studies of HeLa cells, formation of these phosphoinositides and the phosphorylation of Akt were found to be sensitive to PI3-kinase inhibitors LY294002 and wortmannin at only high concentrations, suggesting that kinases other than Class I PI3-kinase are also involved. In contrast, Cooper et al. previously showed that SopB-mediated Akt activation is resistant to 100 nM wortmannin, but completely inhibited by 50 µM LY294002 [20]. However, we believe that such differences in sensitivity may be cell-type dependent, since we saw a significant decrease in SopB-mediated Akt activation when *Salmonella* -infected embryonic stem cells or mouse embryonic fibroblasts were treated with LY294002. Our findings and that of Cooper et al. suggest that the mechanisms of Akt activation by *Salmonella* vary depending on the cell types used for infection.

When Class II PI3-kinase C2β was knocked down in HeLa cells, we observed that SopB-mediated Akt activation was significantly decreased, although this kinase seemed to only play a minor role. No further reduction in Akt activation was noted when such silenced cells were additionally treated with LY294002. We concluded that other kinases must be activated downstream of SopB to induce PI(3,4) P_2_/PI(3–5) P_3_ formation at membrane ruffles. After performing a screen of ~780 host kinases for their potential role in SopB-mediated Akt activation, we identified about 90 kinases that influenced Akt activation after *Salmonella* invasion. This remarkably large repertoire of kinases that contribute to SopB-mediated Akt activation is not surprising, given that SopB is known to be able to manipulate many host kinase phosphorylation events in infected cells [36]. In fact, Rogers et al. have proposed that approximately half of all *Salmonella* -induced phosphorylation events in the host cell require the SopB effector [36]. Manipulation of the phosphorylation status of host kinases may be necessary for SopB-mediated PI(3,4) P_2_ and/or PI(3–5) P_3_ synthesis. It is noteworthy that a complete screen for kinases required for Akt activation has not been previously published. Therefore, the results of our kinase screen are likely to open up new avenues of study into the regulation of Akt.

The kinase that showed the greatest effect on SopB-mediated Akt activation in HeLa cells was IPMK. IPMK is reported to have PI3-kinase activity and it was shown to phosphorylate PI(4,5) P_2_ to generate PI(3–5) P_3_ [37]. Also, IPMK was shown to activate Akt [26]. However, as measured by RT-PCR, IPMK knockdown efficiency was not consistent in HeLa cells, which led to variable extent of reduction in phospho-Akt expression. Thus, we decided to try 2 different knockout cell lines lacking IPMK. To our surprise, we did not see a large decrease in SopB-induced Akt activation in these cells. However, we noted a significant effect of the PI3-kinase inhibitor LY294002 on SopB-induced Akt activation in embryonic stem cells and mouse embryonic fibroblasts, suggesting that Class I PI3-kinases play a more prominent role in these cells. This suggests that SopB can recruit and/or activate various kinases in different cell types to induce PI(3,4) P_2_/PI(3–5) P_3_ formation at invasion ruffles.

How SopB is producing these PIs and exploiting the host kinases needs to be further elucidated. The main substrate of SopB is PI(4,5) P_2_, generating the rare lipid PI(5) P. Since SopB does not seem to have enzymatic activity other than PI-phosphatase activity, the mechanism behind SopB-mediated Akt activation might be via PI(5) P. It is conceivable that PI(5) P recruits distinct lipid kinases to *Salmonella* invasion ruffles to synthesize PI(3,4) P_2_ and/or PI(3–5) P_3_ and/or kinases that directly activate Akt independently of the lipids. In addition, PI(5) P may have an inhibitory effect on protein phosphatases that deactivate Akt, such as PP2A, or on lipid phosphatases that dephosphorylate PI(3–5) P_3_, like SHIP2.

*Shigella flexneri* utilizes a type 3 secreted effector IpgD, a SopB homologue, for infection. Pendaries et al. recently suggested that PI(5) P production by IpgD is necessary for activation of Class I PI3-kinases, thereby activating Akt, in HeLa cells [38]. Although both IpgD and SopB lead to the accumulation of PI(5) P, whether or not SopB-mediated Akt activation is similar to that of IpgD is unclear, because the former is only partially sensitive to PI3-kinase inhibitors in the HeLa cells. It has been shown in vitro that these two homologues have different substrate specificities [14,39]. Hence, a slight difference in the underlying mechanisms of these two bacterial effectors may be possible.

The advantage of bacterial exploitation of several host kinases instead of depending on a single kinase is obvious. Since Akt has been previously shown to play an important role in the *Salmonella* life cycle in the infected host cell, it would be beneficial for the pathogen to manipulate multiple kinases to ensure its activation. Additionally, our data suggests that *Salmonella* may have evolved to use different repertoires of kinases for Akt activation in different types of host cells. This may be intended to adapt to variable expression levels of the kinases in different cell types.

In summary, our data indicate that *Salmonella* can exploit different host kinases during invasion to optimize replication and survival in host cells. SopB generates PI(3,4) P_2_/PI(3–5) P_3_ at invasion sites via a specific group of kinases, which depends on the type of infected host cell. This PI-formation at membrane ruffles is responsible for activating Akt and inhibiting apoptosis in infected cells, as well as modulating SCV biogenesis. However, the precise mechanism of how SopB manipulates different kinases for Akt activation remains incompletely understood and needs further investigation.

## Supporting Information

Figure S1 **SopB expression is sufficient to activate Akt.** HeLa cells were transiently transfected with plasmids encoding untagged SopB, GFP-SopB or GFP. As a control, cells were stimulated with 100 ng/mL EGF for 5 min. Akt activation of cells was assessed 8 h after transfection by immunoblotting the cell lysates with a phospho-specific anti-Ser473 Akt antibody. Pan-Akt antibodies were used to ensure equal protein loading.
(TIF)

Figure S2 **SopB-mediated Akt activation in HeLa cells is only partially sensitive to PI3-Kinase inhibitor Wortmannin.** HeLa cells were treated with different concentrations of Wortmannin (10 nM to 1000 nM) for 30 min. Cells were then infected with wild type *S* *. Typhimurium* for 30 min or incubated with 100 ng/mL EGF for 5 min. As controls, cells were either uninfected or infected with ΔSopB mutant *S* *. Typhimurium* for 30 min. Akt activation was determined by immunoblotting the cell lysates with a phospho-specific anti-Ser473 Akt antibody. Pan-Akt antibodies were used to ensure equal protein loading.
(TIF)

Figure S3 **SopB is not sufficient to produce formation of PI(3–5) P_3_*in vitro*.** Reaction mixtures of FL-PIP_2_ or FL-PIP_3_ and His-tagged recombinant SopB or catalytically inactive SopB (C460S) were separated on TLC. As a control, pure lipids were directly separated on TLC and liposomes without recombinant SopB were also examined.
(TIF)

Figure S4 **IPMK does not contribute to SopB-mediated Akt activation in mouse embryonic fibroblasts (MEF).** (A) Wild type or IPMK knockout mouse embryonic fibroblasts were infected with wild type or Δ*sopB* mutant *S* *. Typhimurium* for 30 min. As controls, cells were uninfected or treated with 100 ng/mL EGF for 5 min. Where indicated, cells were treated with 100 µM LY294002 for 30 min prior to infection. Akt activation was determined by immunoblotting the cell lysates with a phospho-specific anti-Ser473 Akt antibody. Pan-Akt antibodies were used to ensure equal protein loading. (B) Western blot results from A were analyzed by estimating the intensities of protein bands with the ImageJ software. Shown on the graph are the relative and normalized expression levels of phospho-Akt ± SD for three separate experiments, calculated as outlined in the Materials and Methods. The p-values from one-way ANOVA analysis are shown.
(TIF)
