# Supplementary figures and images for: Multiple Host Kinases Contribute to Akt Activation during Salmonella Infection
Source: PLoS One. 2013 Aug 22;8(8):e71015. doi: 10.1371/journal.pone.0071015 (PMC3750030; doi:10.1371/journal.pone.0071015)

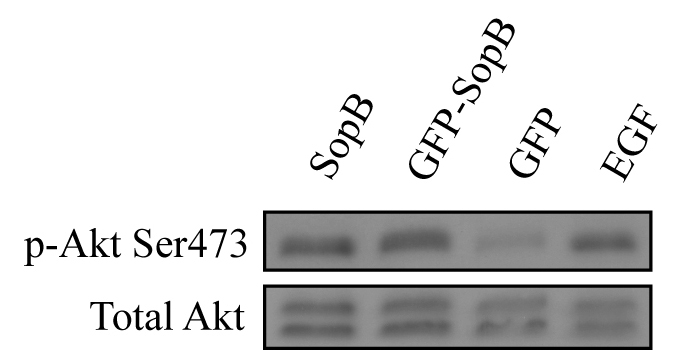

Supplement: Figure S1 — SopB expression is sufficient to activate Akt. HeLa cells were transiently transfected with plasmids encoding untagged SopB, GFP-SopB or GFP. As a control, cells were stimulated with 100 ng/mL EGF for 5 min. Akt activation of cells was assessed 8 h after transfection by immunoblotting the cell lysates with a phospho-specific anti-Ser473 Akt antibody. Pan-Akt antibodies were used to ensure equal protein loading. (TIF) [file pone.0071015.s001.tif]

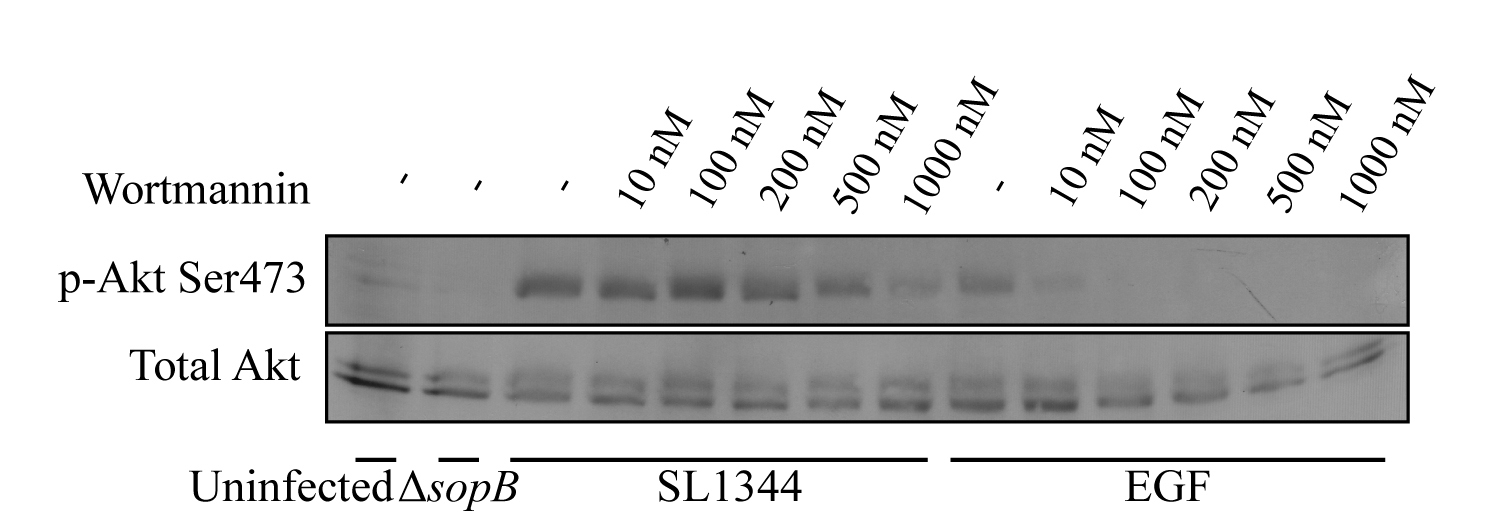

Supplement: Figure S2 — SopB-mediated Akt activation in HeLa cells is only partially sensitive to PI3-Kinase inhibitor Wortmannin. HeLa cells were treated with different concentrations of Wortmannin (10 nM to 1000 nM) for 30 min. Cells were then infected with wild type S . Typhimurium for 30 min or incubated with 100 ng/mL EGF for 5 min. As controls, cells were either uninfected or infected with ΔSopB mutant S . Typhimurium for 30 min. Akt activation was determined by immunoblotting the cell lysates with a phospho-specific anti-Ser473 Akt antibody. Pan-Akt antibodies were used to ensure equal protein loading. (TIF) [file pone.0071015.s002.tif]

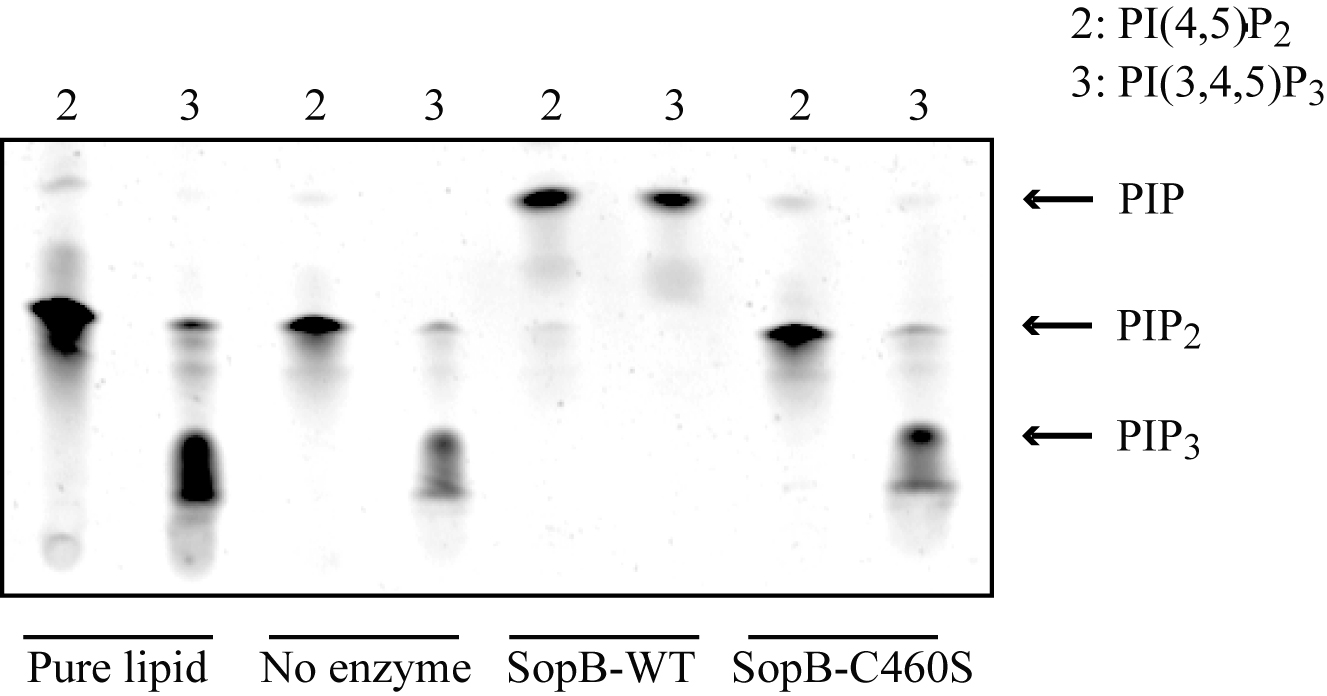

Supplement: Figure S3 — SopB is not sufficient to produce formation of PI(3–5) P3in vitro. Reaction mixtures of FL-PIP2 or FL-PIP3 and His-tagged recombinant SopB or catalytically inactive SopB (C460S) were separated on TLC. As a control, pure lipids were directly separated on TLC and liposomes without recombinant SopB were also examined. (TIF) [file pone.0071015.s003.tif]

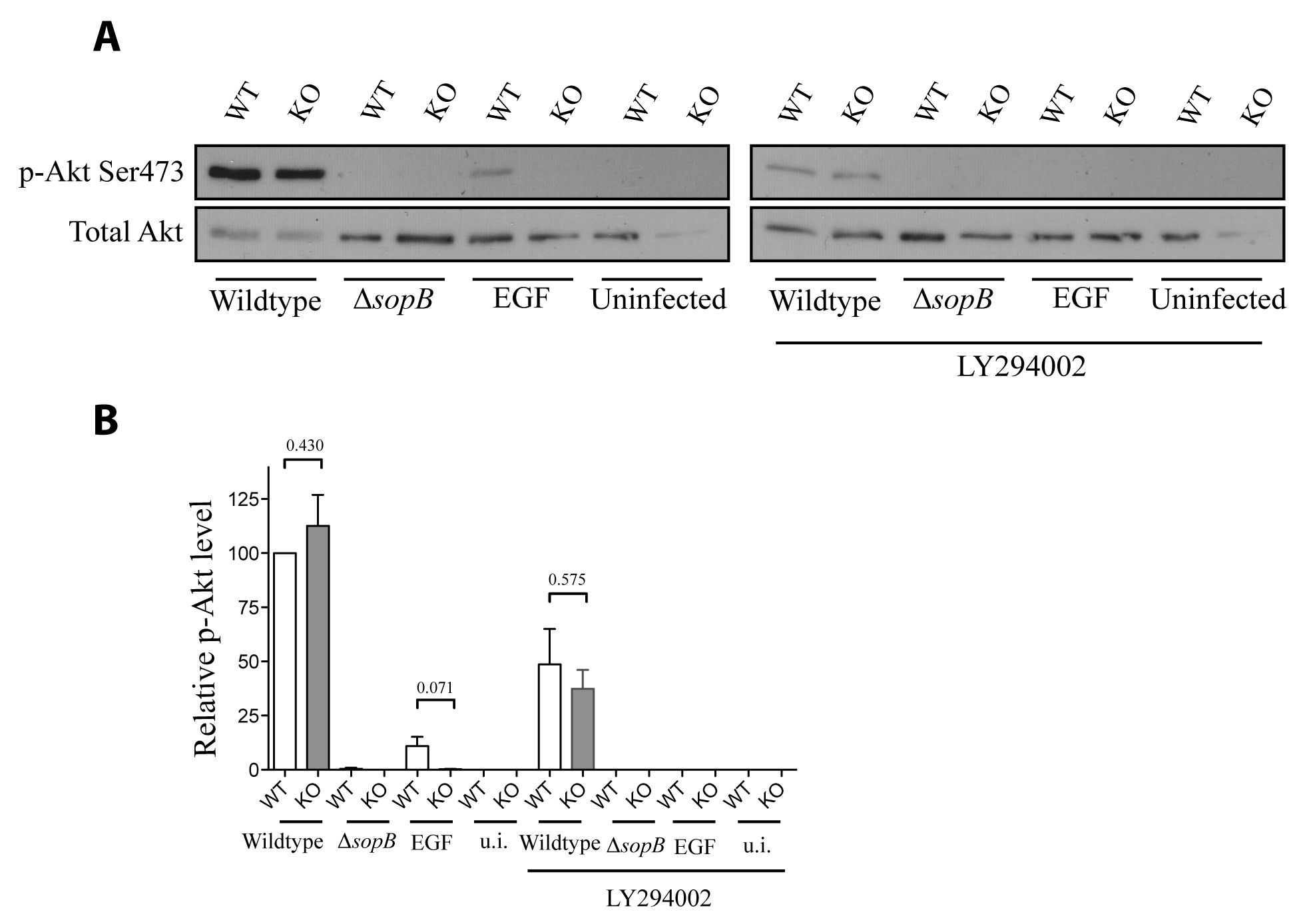

Supplement: Figure S4 — IPMK does not contribute to SopB-mediated Akt activation in mouse embryonic fibroblasts (MEF). (A) Wild type or IPMK knockout mouse embryonic fibroblasts were infected with wild type or ΔsopB mutant S . Typhimurium for 30 min. As controls, cells were uninfected or treated with 100 ng/mL EGF for 5 min. Where indicated, cells were treated with 100 µM LY294002 for 30 min prior to infection. Akt activation was determined by immunoblotting the cell lysates with a phospho-specific anti-Ser473 Akt antibody. Pan-Akt antibodies were used to ensure equal protein loading. (B) Western blot results from A were analyzed by estimating the intensities of protein bands with the ImageJ software. Shown on the graph are the relative and normalized expression levels of phospho-Akt ± SD for three separate experiments, calculated as outlined in the Materials and Methods. The p-values from one-way ANOVA analysis are shown. (TIF) [file pone.0071015.s004.tif]
